# Vegan diets: practical advice for athletes and exercisers

**DOI:** 10.1186/s12970-017-0192-9

**Published:** 2017-09-13

**Authors:** David Rogerson

**Affiliations:** Academy of Sport and Physical Activity, Sheffield Hallam University, S10 2BP, Sheffield, UK

**Keywords:** Vegan, Vegetarian, Plant-based, Diet

## Abstract

With the growth of social media as a platform to share information, veganism is becoming more visible, and could be becoming more accepted in sports and in the health and fitness industry. However, to date, there appears to be a lack of literature that discusses how to manage vegan diets for athletic purposes. This article attempted to review literature in order to provide recommendations for how to construct a vegan diet for athletes and exercisers. While little data could be found in the sports nutrition literature specifically, it was revealed elsewhere that veganism creates challenges that need to be accounted for when designing a nutritious diet. This included the sufficiency of energy and protein; the adequacy of vitamin B12, iron, zinc, calcium, iodine and vitamin D; and the lack of the long-chain *n*-3 fatty acids EPA and DHA in most plant-based sources. However, via the strategic management of food and appropriate supplementation, it is the contention of this article that a nutritive vegan diet *can* be designed to achieve the dietary needs of most athletes satisfactorily. Further, it was suggested here that creatine and β-alanine supplementation might be of particular use to vegan athletes, owing to vegetarian diets promoting lower muscle creatine and lower muscle carnosine levels in consumers. Empirical research is needed to examine the effects of vegan diets in athletic populations however, especially if this movement grows in popularity, to ensure that the health and performance of athletic vegans is optimised in accordance with developments in sports nutrition knowledge.

## Background

Vegan diets might be becoming more visible, owing to the proliferation of social media as a means to share information, experiences and discuss opinions [1]. Promoted by some for alleged health benefits such as reduced risk of heart disease, lower LDL, blood pressure, type II diabetes and cancer [2, 3], veganism is a form of vegetarianism that prohibits the consumption of animal products [4]. Several high-profile athletes, such as former world heavyweight champion boxer David Haye and ladies tennis champion Venus Williams, have reportedly adopted vegan diets in recent times. Quite often, veganism is the product of strong ethical beliefs concerning animal welfare, and vegan activists have been subject to stigma [5], stereotyping [6] and negative attitudes [7], due in part to their vocal denigration of animal consumerism. The increased visibility of high-profile vegan competitors might suggest that veganism could be becoming more appealing for some, especially if more successful athletes adopt and publicize their vegan lifestyles.

Poorly constructed vegan diets however might predispose individuals to macronutrient (protein, *n*-3) and micronutrient (vitamin B12 and vitamin D; iron, zinc, calcium, iodine) deficiencies [2, 3, 8, 9]. This is of particular concern if little attention is paid to accommodating for the nutrients that are excluded due to the elimination of animal products from the diet [9]. Some have alleged that a vegan diet could offer potential performance benefits due to the antioxidant (polyphenols), micronutrient (vitamin C, E) and carbohydrate-rich foods typical of plant-based diets assisting training and enhancing recovery [10, 11]. However, empirical research validating this claim is either equivocal or missing [12]. Indeed, there appears to be a lack of research into veganism in sport in general, despite interest in literature elsewhere [13]. In order to ensure that vegan diets meet both health and performance needs, basic dietary requirements have to be met and sport-specific diet-related objectives need to be achieved [9, 14]. The aim of this article is to address this point, and to provide practical recommendations for sports dieticians, coaches and trainers who might work with vegan athletes. Particular attention will be paid to the achievement of macro and micronutrient requirements for athletic and health-related purposes in this article, as well as a discussion of supplements and ergogenic aids that might be of use to performers who adopt this lifestyle choice.

## Main Text

The information in this narrative has been extrapolated from a broad range of academic disciplines, such as the epidemiological and health sciences, in addition to sports nutrition literature. This is due to little information being available that discusses or investigates veganism in sport and health and fitness-related contexts. Therefore, in some instances, recommendations provided herein have yet to be fully authenticated via scientific investigation, and serve as illustrative concepts until further validation can be undertaken.

### Energy

For most athletes, a well-constructed diet (omnivorous or otherwise) should provide sufficient energy in order to achieve energy balance [15]. However, data suggests that a negative energy balance is common in endurance athletes and athletes participating in weight-making and aesthetic sports (such as combat sports, gymnastics, skating and dancing, etc.) [15]. Very large athletes might also find it difficult to achieve energy balance, particularly during high-volume training phases [16, 17]. Of particular concern in sports that require low body mass, some female athletes might be at risk of developing low bone-mineral density [18]. This is likely to be exacerbated by a poorly-constructed hypocaloric diet [18]. Additionally, high intensity training can reduce appetite [19], and hectic travel schedules, poor food availability (whilst abroad or away from home) and gastrointestinal discomfort might mean that some athletes find it difficult to meet their energy requirements due to various factors [17, 20].

The consequences of insufficient energy are important. Immunity might become compromised, leading to illnesses and time off from training and competition [15, 21]. Weight loss can ensue, and can lead to the loss of muscle mass, reduced strength, lower work capacity and a lack of satisfactory training adaptation [15]. Managing energy balance is thus important for all athletes, but this issue is likely to be compounded further when a habitual diet promotes early satiation and reduced appetite, such as a vegan diet [3, 4, 8–11]. Well-accepted methods of calculating energy intake include estimates such as the Cunningham or Harris-Benedict eqs. [22], Dietary Reference Intakes (DRIs) and/or other literature-based guidelines can all be used to determine nutrient needs and design diets [17]. The International Society of Sports Nutrition (ISSN) recommends that energy requirements should be scaled to activity level, body-mass and mode of exercise [16], to ensure that individual-specific needs are met [17]. Such recommendations are prudent in light of the preceding discussion, as well as the likelihood that athletes possess individual-specific energy and nutrient requirements which differ on the basis of sport, training and competition characteristics [15–17].

Data indicates that vegans consume less energy than omnivores [8], and research suggests that vegetarian diets generally appear to be lower in protein, fat, vitamin B12, Riboflavin, vitamin D, calcium, iron and zinc when compared to an omnivorous diet [8, 14, 23, 24]. Table 1 details vegetarian diets as described in the literature, and highlights how the diets differ based on the extent of their restrictions. Some vegan diets promote the consumption of raw foods only, and data suggests that these diets might lead to poor macronutrient absorption and weight loss when consumed ad libitum [25]. Vegetarian and vegan diets can also lead to very high fibre consumption [14, 24, 25], and plant-based foods therefore tend to have low energy density and promote early satiety [26]. While these factors might be helpful for weight-loss purposes [27], these factors might lead to problems when trying to achieve a high Calorie diet. Where a high Calorie diet is needed, increasing feeding frequency [28] and increasing consumption of energy dense foods such as nuts, seeds and oils [29] might be helpful to ensure that Calorie goals are met. Monitoring and adjusting the diet on the basis of unwanted body mass fluctuations in such cases would also allow for diets to be tailored to individuals’ energy and nutrient requirements [30].

**Table 1:** Vegetarian Diets: Definitions

Type^a^	Description
Flexitarian^b^	Occasionally consumes animal flesh (meat, poultry) and fish, eggs, dairy
Pesco-vegetarian	Excludes animal flesh but does include fish
Lacto-ovo vegetarian	Excludes all flesh; includes diary and eggs only
Lacto vegetarian	Excludes all flesh and eggs; includes dairy only
Ovo vegetarian	Excludes all flesh and dairy; includes eggs only
Vegan	Excludes all animal products
Macrobiotic vegetarian^b^	Variable dietary restrictions; includes wild meat/game and fish in some variations of the diet
Fruitarian	Includes fruit, nuts, seeds and a some vegetables

^a^Definitions from Phillips 2004 [14]

^b^Readers are advised to exercise caution in their interpretation of Flexitarian and Macrobiotic diets as vegetarian diets; owing to their selective inclusion of meat, poultry, fish and seafood, such diets might not be truly vegetarian

### Macronutrients

#### Protein

The role of protein in the athlete’s diet has garnered much attention over the years, and there has been ongoing debate about whether athletes require greater amounts of protein than non-athletic populations [31–33]. The consensus appears to be that athletes require more protein than the lay population [33, 34]. Data also indicates that protein requirements should be tailored to reflect sport-specific and training-goal requirements [35–37]. Typical recommendations therefore include 1.6–1.7 g ∙ kg^−1^ ∙ day^−1^ for strength and power athletes and 1.2–1.4 g ∙ kg^−1^ ∙ day^−1^ for endurance-sport athletes—values notably larger than the 0.8 g ∙ kg^−1^ ∙ day^−1^ recommended for most non-active adults [36, 37]. Values as high as 4.4 g ∙ kg^−1^ ∙ day^−1^ have also been investigated in the literature recently, with favourable body-composition effects noted as a results of its composition [38].

The role of protein in the athlete’s diet is multifaceted. Protein serves as a substrate for exercise performance and a catalyst for exercise adaptation [32]. The balance between Muscle Protein Breakdown (MPB) and Muscle Protein Synthesis (MPS) is known as Net Protein Balance (NPB). Achieving a positive NPB via elevated MPS promotes exercise recovery, adaptation and anabolism [32, 38, 39]. During negative energy balance adaptive mechanisms preserve Fat Free Mass (FFM) under hypocaloric conditions [33, 40]. Despite this, dieting athletes and bodybuilders might still require elevated protein intakes due to the need to preserve lean mass and promote satiety [33, 39]. Concurrent resistance and endurance training might also compound the need for extra protein during a hypocaloric diet [33, 39]. Athletes involved in weight-categorised and aesthetic sports need to be cognisant of optimizing protein intakes, where the preservation of FFM and optimization of relative strength is likely to be advantageous to performance. The ISSN provides a broad protein recommendation of 1.4–2.0 g ∙ kg ∙ day^−1^, which is likely to be appropriate for most athletic contexts [34]. However, for athletes in need of losing body-mass, recommendations of up to 1.8–2.7 g ∙ kg ∙ day^−1^ have been provided in literature [33, 39], and values as high as 2.3–3.2 g ∙ kg ∙ FFM ∙ day^−1^ have been suggested for bodybuilders aiming to achieve competition-level leanness [39].

Vegan athletes however appear to consume *less* protein than their omnivorous and vegetarian counterparts [11]. The optimisation of protein intakes for vegan athlete requires that attention is paid to the quantity *and* quality of protein consumed [41]. Plant-based protein sources are often incomplete, missing important essential amino acids, and typically contain less Branched Chain Amino Acids (BCAA) than their animal-based equivalents [34, 35]. Leucine appears to be a primary trigger of MPS, and plays an important role in promoting recovery and adaptation from exercise [32, 34, 41]. Interestingly, evidence suggests that milk-based proteins might be superior to other protein sources at promoting MPS, mediated in part by the richness of its BCAA content [42, 43]. Similarly, the habitual consumption of milk as part of a diet and resistance-training programme might lead to better muscle hypertrophy when compared to a soy-protein-supplemented equivalent [44, 45]. This is might be due to milk’s superior amino acid composition [45]. Indeed, plant-based proteins often lack essential amino acids [46], and animal-based proteins therefore possess a greater biological value due to the presence of all essential amino acids in the food [46]. Common examples of the limiting amino acids in plant-based proteins include lysine, methionine, isoleucine, threonine and tryptophan. Of these, lysine appears to be to be most commonly absent, particularly from cereal grains [46]. Foods such as beans and legumes are rich sources of lysine however, and leucine can be obtained from soy beans and lentils. Other BCAAs can be found in seeds, tree nuts and chickpeas, meaning that these amino acids can be obtained by consuming a variety of protein-rich, plant-based foods [14, 46]. Indeed, the Academy of Nutrition and Dietetics (AND) have recommended that a range of plant-based proteins should be consumed by vegetarians in order to meet their protein and amino acid requirements [47]. Further, the once-popular recommendation of combining protein sources to achieve a complete essential amino acid profile in each feeding is no longer considered necessary [14]. Foods such as grains, legumes, nuts and seeds should be included in the vegan diet to ensure that all EAAs are present, and that adequate BCAA are consumed to support recovery and adaptation from training. Examples of high-protein vegan-friendly foods can be found in Table 2.

**Table 2:** High Protein Foods

Food	Protein per 100 g^a^
Pumpkin seeds (dried, uncooked)	30.2
Lentils (red, split, uncooked)	24.6
Black beans (uncooked)	21.6
Almonds (raw)	21.2
Tempeh	20.3
Tofu (calcium set)	17.3
Oats (rolled)	16.9
Quinoa (uncooked)	14.1

^a^Data from USDA food composition database SR28

Plant-based protein supplements that feature in the literature and are commercially available include soy (and soy isolate), pea, rice, hemp and composite/blended protein products [45–48]. Supplemental protein might be of interest to vegan athletes, particularly if achieving sufficient protein via wholefoods is either difficult or inconvenient. Emerging data is beginning to support the efficacy of plant-based-protein powders at improving recovery from training [48] and fostering muscle hypertrophy as part of a resistance training program [45]. Recent evidence also suggests like-for-like responses when comparing supplemental plant and dairy proteins on body composition and exercise performance as part of a training programme [48], contrasting previously-reported data [45]. In comparison to dairy-based protein supplements however, plant-based supplements appear to be much less researched at this time, and further research is needed to understand the effects of individual (rice, pea, hemp, etc.) and blended products on postprandial MPS [49].

#### Protein digestibility

The digestibility of plant-based protein appears to be markedly less than that of animal products, which might need to be accounted for when designing a vegan diet [50]. The Protein Digestibility Corrected Amino Acid Score (PDCAAS) and Digestible Indispensable Amino Acid Score (DIAAS) are metrics that rate the quality of proteins based on their digestibility [51]. The PDCAAS has been criticised for disregarding anti-nutrient factors that affect protein absorption, and for truncating protein sources that score in excess of its 1.0 ceiling [51]. The DIAAS does neither, and is perhaps a superior system for rating protein digestibility [41, 51]. Both systems however indicate that animal-derived proteins score higher than plant-based sources [51]. Interestingly, soy protein possesses a PDCAAS of 1.0 and appears to be comparable to whey protein isolate. However, when factoring in anti-nutrient factors such as phytic acid and trypsin inhibitors, which limit the absorption of nutrients, whey protein isolate appears to be superior to soy protein when using the DIAAS (1.09 vs. 0.91) [41]. Other important plant-based protein sources such as rice, peas and hemp all score markedly lower than animal-based sources such as eggs, chicken and beef using either system [41, 51, 52]. Indeed, it has been suggested that vegetarians might need to consume *more* protein than meat eaters to compensate for the poorer digestibility of plant-based sources [50]. Values of up to 1.0 g ∙ kg^−1^ ∙ day^−1^ (vs. the RDA’s 0.8 g ∙ kg^−1^ ∙ day^−1^) have been suggested for a non-athletic, vegetarian population, who might consume eggs and dairy products in addition to plant-based proteins [50]. Due to the absence of all animal proteins in the diet, it might be prudent for vegan athletes to aim for protein intakes towards the higher end of the ISSN’s protein recommendation of 1.4 to 2.0 g ∙ kg ∙ day^−1^, whilst in an energy-neutral or energy-positive state. In some instances, values of up to 1.8 g ∙ kg^−1^ to 2.7 g ∙ kg ∙ day^−1^ might be appropriate during weight loss phases, to compensate for the reduced digestibility and low biological value of plant-based sources [33, 39].

#### Carbohydrate

Vegan diets tend to be higher in carbohydrates, fibre, fruits, vegetables, antioxidants and phytochemicals than omnivorous diets [53]. The consumption of micronutrient and phytochemical-rich foods is an important benefit of any plant-based diet [3, 9]. This might help to mitigate the effects of excess inflammation and promote recovery from training, although this has yet to be confirmed empirically [10, 12]. It has been suggested that some endurance athletes might intentionally adopt a vegan diet in order to meet their carbohydrate needs, or to assist weight management goals [10, 11, 54]. Carbohydrate requirements in sport has been the focus of literature debate for some time [55], and athletic diets generally require carbohydrate intakes of 4 to 12 g ∙ kg^−1^ to support high training volumes, depending on the mode of exercise, the athlete’s gender and goal of the athlete’s diet [17].

Achieving an adequate carbohydrate intake via a vegan diet is relatively straightforward, and grains, legumes, beans, tubers, root vegetables and fruits can all be consumed to meet carbohydrate requirements satisfactorily. In order to achieve sufficient protein via the consumption of whole foods as recommended in this article, it is recommended that vegans consume beans, pulses, lentils and grains daily—foods that are also abundant in carbohydrate. However, recall that these foodstuffs are rich sources of fibre. Fibrous, non-digestible carbohydrates and lignin provide volume and bulk, are resistant to digestion and absorption, and promote early satiation and enhance prolonged satiety signalling [47, 56, 57]. For athletes requiring higher energy intakes, the consumption of fibre-rich foods to achieve protein and carbohydrate adequacy might prove to be difficult for some. Due to the lectins in foods such as beans, grains, nuts and potatoes [58], as well as the fermentation of resistant starch and indigestible carbohydrates (found in oats, peas, beans, fruits, and in certain vegetables and lentils), a high-fibre diet can also promote gastric distress in some cases [38, 59, 60]. In order to achieve sufficient carbohydrate for the athletes involved in high-volume training phases it might be appropriate (in some contexts) to choose *some* lower-fibre foods when developing high-carbohydrate meals, as long as sufficient micronutrient status (particularly B vitamins) can be ensured. Foods such as rice, pasta, noodles and buckwheat contain less fibre than oats, lentils, beans and wholegrain breads, and removing the skin from tubers and root vegetables reduces the fibre contents of these foods whilst maintaining decent carbohydrate levels.

#### Carbohydrate timing and supplementation

The optimisation of carbohydrate consumption with respect to training and competition has been debated in the literature [61]. Conventional wisdom suggests that maintaining high muscle glycogen stores (achieved via a carbohydrate-rich diet), consuming carbohydrates before and during exercise (scaled to absolute intensity and mode of exercise), consuming multiple-transportable carbohydrates (such as a glucose-fructose mixture), and carbohydrate mouth rinsing (to delay fatigue) might enhance performance during middle-distance and endurance events [37, 62]. The effects of carbohydrate consumption before and during short-duration high-intensity sports are equivocal [36], however carbohydrate feedings 0–60 min prior to exercise have been indicated for events lasting >30 mins [37, 62]. Carbohydrate doses during activity can be scaled based on the event, where more/less carbohydrate is consumed with respect to the time and intensity of sport/exercise performance [37, 62]. In most cases, carbohydrate supplements appear to be vegan-friendly, and so their consumption is feasible for most vegan athletes. Consuming calcium-fortified fruit juices as a liquid carbohydrate might serve dual purposes however, and enable vegans to meet both carbohydrate and calcium needs whilst concomitantly offering possible ergogenic advantages if used as indicated in the literature [61].

#### Fat

Vegan diets are typically lower in total and saturated fat and higher in *n*-6 fats than omnivorous and vegetarian diets [8, 13, 63]. This trend appears to be associated with reductions in heart disease, hypertension, type II diabetes, cholesterol and cancer [63], and is a purported health benefit of veganism. However, the role of fat in the diet is an area of much discussion, and deleterious effects of fat consumption are not universally accepted [64–66]. Indeed, in some cases, high-fat diets have even been promoted [55]. Interestingly, research has indicated that low-fat dieting might negatively influence testosterone levels in males [67]. This might be of interest to athletes needing to maximise anabolism and adaptation to resistance training. However, despite reporting lower total and saturated fat intakes, evidence has also suggested that vegan males do not have statistically lower androgen levels than omnivores [68]. Relationships between fat consumption, hormones and sport performance might require additional investigation. In many instances, it appears that the health implications of a dietary fat might reflect its fatty acid composition [64, 69], meaning that attention should be paid to the quantity and quality of fat consumed. Achieving recommended values of 0.5–1.5 g ∙ kg ∙ day^−1^ (or 30% of daily caloric intake) is feasible for vegan athletes given adequate consumption of oils, avocados, nuts and seeds.

#### ALA, EPA and DHA

Due to an absence of marine-sourced fats, vegans appear to consume fewer *n*-3 fatty acids and possess lower serum *n*-3 fatty acid levels than omnivores and other vegetarians [8, 13, 63, 70]. This might have important health and performance implications. The *n*-3 fatty acids are important for normal growth and development, and appear to play an important role in cardiovascular health [71], in inflammatory and chronic disease [72], and might improve exercise-induced bronchoconstriction (EIB) and immunity [73]. Of interest to athletes, *n-*3 fats might also increase nitric oxide production [71, 74], and improve heart-rate variability [75]. Both *n*-6 and *n*-3 fatty acids are parent fatty acids for eicosanoids (prostaglandins, thromboxanes and leukotrienes), and *n*-3 fatty acids appear to possess anti-inflammatory, antithrombotic, antiarrhythmic; hypolipidemic, vasodilatory and antiproliferative properties [71, 72]. Both *n*-6 and *n*-3 fatty acids are essential, however the long chain *n*-3 fatty acids eicosapentaenoic acid (EPA) and docosahexaenoic acid (DHA) are considered to be under-consumed in the modern western diet in general [72], and in vegans in particular [47, 70].

There is ongoing debate about the quantity and/or ratio of *n-*3 to *n*-6 needed to manipulate the synthesis of pro and anti-inflammatory eicosanoids in order to impact health and performance favourably [76–78]. In the UK, an upper limit of 10% of energy from pro-inflammatory *n*-6 fatty acids has been recommended by the Department of Health to reduce negative effects of overconsumption [78]. Elsewhere, Sanders [79] and Philips [14] recommend that vegetarian diets limit linoleic acid consumption (an *n-*6 fatty acid), found in sunflower, corn and safflower oils, for similar purposes. The *n-*3 α-linolenic acid (ALA) is an important constituent of cellular membrane and is converted to EPA at ~8% efficiency in humans, which appears to be both age and gender-specific in magnitude [80]. Roughly 0.5% of ALA is converted to DHA, highlighting humans’ poor ability to enzymatically convert ALA to this fatty acid [81]. While humans *do* convert a small amount of ALA to DHA, the primary source of this in the diet is cold water fish and seafood. EPA and DHA exert many of the reported health and performance benefits of *n*-3 fatty acid consumption and is now a popular supplement [80]. Supplemental ALA has been shown to increase blood EPA levels [82] but does not appear to affect DHA status [83]. Microalgae oil is rich in DHA (and EPA) and might be a useful supplement for vegans and vegetarians. Microalgae-oil supplements have been shown to raise both blood EPA and DHA levels [84]. However, recommendations for vegan-friendly DHA supplements do not appear in the literature at this time [9]. Recommendations *do* appear for other food sources of the *n*-3 ALA, such as flax seeds, walnuts, and chia seeds [9, 14]. Interestingly, flax/linseeds are also rich in lignan precursors, which might offer broader health-related benefits [85], and chia is also a complete protein [86]. Combining whole-food sources of ALA as indicated in this article with supplemental DHA derived from microalgae oil might optimise a vegan’s *n*-3 fatty acid intake, and improve health concurrently with any health and performance-enhancing effect that augmented *n-*3 diets might offer athletes [76, 77]. Research detailing how to optimise *n-*3 consumption for vegans is missing at the time of writing; however, recommendations of 1–2 g ∙ day^−1^ of combined EPA and DHA at a ratio of 2:1 have been suggested for athletes elsewhere [77]. To achieve a DHA dose of 500 - 1000 mg ∙ day^−1^, this would equate to 1–2 g of microalgae oil, or 2–4 capsules in most commercial products.

### Micronutrients

Achieving micronutrient sufficiency is an important concern for all athletes. The AND have indicated that attention should be paid to achieving adequacy in vitamin B12, iron, zinc, calcium, iodine and vitamin D intakes when designing a vegan diet in particular [47]. Poorly designed diets might predispose individuals to deficiency regardless of predilection, which could have detrimental health and performance implications [2, 9, 12]. This needs to be understood by those seeking to adopt veganism, and strategies to mitigate the risks of under-consuming these nutrients need to be present if a vegan diet is to optimize health and performance. Table 3 compares the nutritional implications of several diets (omnivorous, pesco-vegetarian, vegetarian and vegan), and provides recommendations for athletes and practitioners. The following section will identify and elaborate upon concerns highlighted in the literature, based upon research indicating what micronutrients might be under-consumed in a vegan diet [2–4, 8, 14, 47, 63, 70, 87].

**Table 3:** Diet Comparison

Diet type	Possible dietary Issues^a^	Possible sport-related issues^a^	Recommendations^b^
Omnivorous	Poor ad libitum diets can lead to nutrient deficiency. Vitamin D deficiency possible (if sun exposure is poor / unlikely).	Male and female athletes with low energy intake at risk of nutrient deficiencies. Calcium requirements increased during negative energy balance, amenorrhea and female athlete triad.	Energy intake should be scaled to activity level. Depending on sport, 1.4–2.0 g ∙ kg^−1^ protein; 3–10 g ∙ kg^−1^ CHO; 0.5–1.5 g ∙ kg^−1^ fat (or, 30% energy) consumed daily. Micronutrient-rich diet sufficient to achieve DRVs; Vitamin D3 supplement might be necessary.
Pesco-vegetarian	Same as omnivores plus: Energy^c^, protein.	Iron deficiency with and without anaemia a risk in female athletes.	Same as omnivores, plus ensure that iron needs are met via a variety of food sources.
Lacto-ovo vegetarian & Lacto-vegetarian	Same as pesco-vegetarians plus: Long chain *n-*3 (EPA, DHA), iron, zinc, riboflavin deficiencies more likely.	Same as pesco-vegetarians plus: Reduced muscle creatine and carnosine stores a possibility in males and females.	Same as pesco-vegetarians plus: EPA / DHA supplement (total 1–2 g ∙ day^−1^; 2:1 ratio) might be needed. Increase iron (m = 14 mg & f = 33 mg ∙ day^1^) and zinc (16.5 mg & 12 mg ∙ day^1^) intakes due to reduced bioavailability of plant sources.
Vegan	Same as vegetarians plus: Protein, fat, *n*-3, B12, calcium, iodine deficiencies also possible / likely in males and females.	Same as vegetarians plus: Low bone-mineral density is an increased possibility in female athletes. Achieving energy balance might be a problem for larger athletes.	Same as vegetarians plus: Increase protein to 1.7–2.0 g ∙ kg^−1^ and up to 1.8–2.7 g ∙ kg^−1^ during weight loss phases (obtain from range of plant-based foods). Nuts, seeds, avocados, oils to achieve 0.5–1.5 g ∙ kg^−1^ fat daily. EPA / DHA (microalgae); vitamin D3 (lichen) & B12 supplements might be needed; iodine in some instances too. 1000 mg ∙ day^−1^ calcium from beans, pulses, fortified foods and vegetables.

^a^Data from various sources [8–11, 13, 14, 23–25, 47, 63, 70, 87]

^b^Recommendations from various sources [9–11, 16, 17, 22, 47]

^c^Energy balance a potential issue in endurance, weight-making and aesthetic sports and larger athletes regardless of diet [15]

#### Vitamin B12

Due to an absence of animal and dairy products, vegans are at an increased risk of developing Vitamin B12 (cobalamin) deficiency [87]. Cobalamin is synthesised from anaerobic microorganisms, in the rumen of cattle and sheep, and humans typically consume pre-formed cobalamin from animal products, which are the main source of B12 in the diet [88]. Plant-based sources of cobalamin are unusual, unless the plant has been contaminated by manure or from animal waste [47, 88]. Cobalamin is essential for normal nervous system function, homocysteine metabolism and DNA synthesis [88]. Insufficient cobalamin can lead to morphological changes to the blood cells and the development of haematological and neurological symptoms, such as megaloblastic anaemia and neuropathy [89]. Long-term cobalamin deficiency can lead to irreversible neurological damage, and data indicates that veganism can lead to deficiency if cobalamin is not supplemented [14]. Data from the EPIC-Oxford cohort study in the UK indicated that ~50% of vegan participants were vitamin B12 deficient [90]. An additional 21% of the vegans were also classified as having very low levels. Interestingly, despite 20% of participants consuming a B12 supplement, blood-vitamin levels between those that did vs. those that did not take supplements were no different, suggesting that the supplementation practices of the cohort were inadequate to achieve B12 sufficiency. Sources of vitamin B12 suitable for a vegan diet include B12-fortified breakfast cereals and nutritional yeast, as well as dietary supplements. Supplemental vitamin B12 products typically contain cyanocobalamin, although other forms such as methylcobalamin and hydroxocobalamin are available—the latter by prescription only. The body appears to have a limited capacity to absorb vitamin B12 supplements orally [88, 89], which is limited by the presence of intrinsic factor, a glycoprotein secreted by the stomach’s parietal cells that combines with B12 prior to absorption in the distal ileum via receptor-mediated endocytosis [89]. For an ingested 500 μg oral supplement, only an approximated 10 μg might be absorbed [89]. Because of this poor bioavailability, sublingual drops, lozenges and transdermal products have been developed and marketed under the pretence that they offer better absorption, however research supporting these claims could not be found when writing this article. The requirement for vegans to supplement with vitamin B12 is important, and vegans are advised to consume fortified foods and/or take a daily supplement to ensure an adequate intake of the vitamin [9, 14]. The Dietary Reference Intake (DRI) for vitamin B12 is 2.4 μg ∙ day^−1^ for adults of both sexes [91], and vegans have been advised to consume up to 6 μg ∙ day^− 1^ of supplemental B12 by some authors [10]. Where adequate B12 status cannot be achieved via oral supplementation and fortified food products alone, vegans might need to have serum levels monitored by a medical practitioner if deficiency is suspected [87]; subcutaneous or intramuscular injections might even be indicated in some contexts [87]; monitoring B12 status carefully might be necessary for some vegan athletes.

#### Iron

The iron status of vegetarians and vegans has received attention in the literature [92–94], and it appears that owing to a diet rich in whole-grains and legumes, both vegetarians and vegans consume similar amounts of iron as omnivores [9, 63]. However, issues with the bioavailability of plant-based iron might mean that vegans need to pay attention to ensuring that sufficiency is prioritized [92, 93]. The main source of iron in the vegan diet is found in the non-haem form, which is less bioavailable than the haem iron found in animal products [93]. Vegan diets also commonly contain dietary inhibitors such as the polyphenols tannin (found in coffee, tea, and cocoa) and phytates (found in whole grains and legumes), which reduce the amount of iron absorbed from the diet. Research into the iron status of vegans has found that female vegans appear to have lower iron stores than omnivores, and are more prone to iron-deficiency anaemia [63, 94, 95]. Male vegans appear to have a similar iron status as non-vegans and are less impacted by iron status [63]. Iron-deficiency anaemia is caused by insufficient consumption of iron (or insufficient absorption of iron) and is a decrease in red blood cells (RBCs) or haemoglobin, leading to symptoms such as tiredness and fatigue; weakness, shortness of breath and reduced exercise tolerance [95]. Iron deficiency *without* anaemia has also been shown to reduce endurance capacity, increase energy expenditure and impair adaptation to endurance exercise in females experiencing tissue depletion [96]. Supplementation has been shown to correct such problems and might be warranted if adequacy cannot be achieved via the diet (97). Indeed, achieving an iron-sufficient diet appears to be rudimentary for *all* female athletes [95–97].

Interestingly, however, it has been suggested that the body can regulate iron absorption based upon blood concentrations of the mineral [14]. Low iron status can lead to intestinal adaptations that increase absorption and reduce secretion in order to maintain equilibrium [14]—an effect that appears to be present with other important micronutrients discussed in this article [3, 97]. It appears that humans can adapt to a wide range of iron statuses and intakes, and vegetarians and vegans generally do not appear to suffer adverse health effects because of reduced iron absorption [98]. Hunt [93], however, recommends that iron intakes for vegetarians be increased by 80%, so that adult males and females achieve a recommended intake of 14 mg ∙ day^−1^ and 33 mg ∙ day^−1^ (vs. the Recommended Daily Allowance’s 8 mg ∙ day^−1^ and 18 mg ∙ day^−1^), due to the aforementioned bioavailability issues. The Institute of Medicine (IOM) concur, and suggest that iron requirements for vegetarians are 1.8 times greater than omnivores [92]. Elevated intakes of iron for vegetarians and vegans have been refuted however on the basis that high iron intakes might increase susceptibility to heart disease and cancer [99], and that supplemental iron might affect the bioavailability of other minerals and copper [14]. Indeed, it has also been suggested that such recommendations have exaggerated requirements by basing recommendations off of acute feeding studies, where the effects of iron inhibitors and enhancers might have been artificially pronounced [3, 92, 100]. Non-haem iron absorption can be enhanced (as well as inhibited), and consuming non-haem iron-rich foods in conjunction with vitamin C appears to increase absorption [9, 92]. Vegan athletes should therefore look to achieve iron sufficiency by choosing wholefood iron sources, reducing their consumption of inhibitor-containing foodstuffs such as tea, coffee and cocoa (when eating iron-rich meals), consume vitamin C containing foods concurrently to enhance absorption, and incorporate soaked, sprouted and/or fermented foods in their diets, if palatable. In cases of where individuals might be prone to iron deficiency, i.e. females with large menstrual blood losses, monitoring iron status and considering supplementation might be necessary. A list of food sources for iron and other nutrients discussed in this article can be found in Table 4.

**Table 4:** Vegan-Friendly Food Sources

Nutrient	Vegan-friendly sources
Protein	Pulses, grains, legumes, tofu, quinoa, nuts, seeds, vegetables
ALA	Flax seeds, walnuts, chia seeds, hemp seeds
EPA^a^	Seaweed, algae
DHA	Microalgae oil, seaweed
Vitamin B12	Supplements, fortified foods, plant milks, nutritional yeast (fortified), fermented soy^b^, mushrooms^b^
Iron	Legumes, grains, nuts, seeds, fortified foods, green vegetables
Zinc	Beans, nuts, seeds, oats, wheat germ, nutritional yeast
Calcium	Tofu (calcium set), fortified plant milks and juice, kale, broccoli, sprouts, cauliflower, bok choi
Iodine	Seaweed, cranberries, potatoes, prunes, navy beans, iodized salt
Vitamin D	Lichen-derived D3 supplements

^a^EPA can also be enzymatically converted from ALA and retroconverted from DHA [83, 84]

^b^Might not be a reliable source of this nutrient

#### Zinc

Zinc is a constituent of enzymes involved in metabolic processes that relate to DNA stabilisation and gene expression, and is important in cell growth, repair and protein metabolism [92]. Similar to iron, zinc is widely available in plant-based foods but is also not readily absorbed [93]. Similarly as well, the body appears to adapt to lower intakes of zinc by reducing losses and increasing absorption in order to maintain equilibrium [3, 97]. It has been suggested therefore that vegetarians do not need to pay special attention to consuming this mineral [3]. However, the IOM have suggested that vegetarians might need to consume up to 50% more zinc than non-vegetarians owing to its poor bioavailability [92]. Indeed, common vegan sources of zinc include beans, whole grains, nuts and seeds (Table 4)—foods that also contain phytate [93]. However, processing foods can reduce phytate too. Leavening bread activates phytase, breaking down phytic acid, and soaking, fermenting and sprouting nuts and grains can all reduce phytate levels and increase nutrient bioavailability [101]. Based on the IOM’s suggestion, it has been recommended that male vegans consume up to 16.5 mg ∙ day^−1^ of zinc (vs. the RDA of 11 mg ∙ day^−1^) and females up to 12 mg ∙ day^−1^ (vs. 8 mg ∙ day^−1^) [10]. Zinc bioavailability appears to be enhanced by dietary protein and inhibited by supplemental folic acid, iron, calcium, copper and magnesium, but might not be affected by the whole-food sources of these nutrients [101]. In order to achieve the above recommendations, vegans should look to consume zinc-rich foods such as hemp and pumpkin seeds, and other grains, nuts and beans (Table 4), and look to adopt processing methods that improve mineral absorption, such as soaking and fermenting, as suggested earlier. If this is not achievable, a supplement should be considered. Owing to issues concerning bioavailability, zinc supplements should not be consumed at the same time as supplemental forms of the aforementioned minerals. Multivitamin/mineral formulas might be inadequate if to ensure nutrient adequacy in this instance.

#### Calcium

Calcium is abundant in a wide range of foodstuffs, most notably dairy products. Data indicates that vegans consume less calcium than omnivores and other vegetarians [63]. Indeed, Canadian vegans have been shown to consume only 578 mg ∙ day^−1^ compared with the 950 mg ∙ day^−1^ and 875 mg ∙ day^−1^ of omnivores and ovo-lacto vegetarians [102]. Vegans have been shown to be at a higher risk of fracture due to lower calcium intakes [103]. Low intakes of calcium are particularly problematic for children and teenagers, where higher calcium requirements are required for bone development [78, 104]. As with other minerals, the body appears to be able to regulate calcium status during periods of low consumption. When habitual calcium intakes are low, and when sufficient vitamin D is present, an increased proportion of calcium is absorbed from food [104]. It has been suggested that lower protein intakes typical of a vegan diet might contribute to greater calcium retention due to high-protein diets promoting calcium excretion in the urine [104, 105]. However, evidence demonstrates that protein-rich diets have *no* effect on calcium retention [105], and in some instances work synergistically with calcium to *improve* calcium retention and bone metabolism [34, 105]. It is widely recommended that adequate calcium is necessary for blood clotting, nerve transmission, muscle stimulation, vitamin D metabolism and maintaining bone structure [106]. Indeed, the importance of calcium for the vegan athlete reflects its role in the maintenance of skeletal health during weight-bearing exercise, and increased calcium losses experienced during heavy perspiration [107]. Calcium requirements might also be exacerbated during phases of calorie restriction, amenorrhea and in some instances of the female athlete triad [107]. However, it is also proposed that the RDA for calcium (1000 mg ∙ day^−1^) is sufficient to meet the requirements of athletic populations in most contexts, and so despite the aforesaid factors, it has been suggested that athletes do not have an elevated requirement for the nutrient in general [107].

In order to meet the above requirement, vegan athletes should consume plant-based sources of calcium such as beans, pulses and green vegetables in sufficient quantities to achieve the 1000 mg ∙ day^−1^ recommendation [106]. Broccoli, bok choy and kale are particularly high in calcium; green vegetables such as spinach and arugula contain oxalate however, which impedes calcium absorption [104]. Vegans therefore should choose plant sources that contain low oxalate levels when designing calcium-rich meals. Calcium-fortified foods are also widely available, and examples such as calcium-fortified soy, nut milks and fruit juices are all vegan-friendly and provide readily absorbable forms of the nutrient (Table 4). Vegans can also consume calcium-set tofu, which is also rich in protein, to help achieve their requirements if palatable. If a vegan diet cannot achieve sufficient calcium levels, then a supplement might also be required [14].

#### Iodine

Iodine is an essential trace element needed for physical and mental growth and development, and plays in an important role in thyroid function and metabolism [92]. Excessively high or low intakes of iodine can lead to thyroid dysfunction, and vegans have been shown to consume both excessively high and low intakes depending on their dietary choices [108, 109]. To highlight, a study by Krajcovicova-Kudlackova and colleagues [110] found that 80% of Slovakian vegans were iodine deficient. Lightowler and Davies [109] however found that some vegans consumed excess iodine (from seaweed) in their study. Common iodine sources include fish and dairy products, and the DRI for Iodine has been set at 150 μg ∙ day^−1^ for adults [92]. Iodine content in foods vary according to the soil-iodine content (when growing produce), the farming methods used during production, the season it is grown in, and the species of fish (if non vegan) [111]. Goitrogens, found in cruciferous vegetables such as cabbage, cauliflower and rutabaga decrease iodine utilisation and might affect adversely thyroid function if consumed in large amounts [111]. However, cooking such foods appears to destroy many of the goitrogenic compounds present, making this effect unlikely. Raw-food vegans should look to limit the consumption of raw, goitrogenic foods where possible.

Seaweed and sea vegetables are a concentrated source of iodine that are vegan-friendly. Excessively high iodine intakes have been reported in vegans who regularly consume seaweed however [109, 112], and in some cases have led to elevated Thyroid Stimulating Hormone (TSH) levels [113, 114]. Elevated TSH might reflect iodine-induced hyperthyroidism or iodine-induced hypothyroidism [114]. However, iodine intakes above 150 μg ∙ day^−1^ appear to be well tolerated, unless clinical susceptibilities to thyroid issues are present [115]. Iodine concentrations in seaweed can vary markedly [115], and the British Dietetic Association suggests that seaweed might not be a reliable iodine source [116]. Iodized table salt has however been indicated for vegans looking to achieve sufficient intakes [14], and iodine can be also found in foods such as potatoes, breads (in some countries) and cranberries (Table 4). Examples of how to achieve sufficient iodine levels (and for other nutrients discussed in this article) can be seen in Tables 5 and 6, which provide menus based on a 2500 and 3500 Calorie requirement. Where iodine sufficiency cannot be achieved through food alone, a supplement that meets the 150 μg ∙ day^−1^ recommendation might be advisable.

**Table 5:** Sample 2500 Calorie menu^a^

Meal	Ingredients
Breakfast: *“Nutty Banana Oatmeal”*	• Oats, ½ cup (uncooked) • Almond milk, fortified, unsweetened, 1 cup • Banana, 1 small (60 g) • Brazil nuts, 1nut • Flax seeds, milled, 1 tablespoon • Pumpkin seeds, 1 tablespoon • 1000 IU Vitamin D3 (lichen derived) • 2.4 μg Vitamin B12 • 2 g microalgae oil supplement
Lunch / Pre-training: *“Asian-style Kale Salad”*	• Kale, 1 cup, chopped • Carrot, 1 medium, shredded • Cucumber, ½ cup, shredded • Dried wakame, 1 g, sprinkles • Rice noodles, 57 g (uncooked weight) • Edamame beans, ½ cup • Tofu, calcium-set, ½ cup • Ginger, garlic, chili, to taste • Sesame oil, 1 tablespoon
Post Training: *“Mixed berry protein smoothie”*	• Pea protein isolate, 40 g • Frozen raspberries, ½ cup • Frozen strawberries, ½ cup • Frozen blueberries, ½ cup • Hemp milk, fortified, 1 cup
Dinner: *“Garbanzo and Sweet potato Curry”*	• Garbanzos, 120 g, cooked • Seitan, 40 g • Sweet potatoes, 1 medium • Tomatoes, ½ can • Onion, 1 red, small • Garlic, 1 clove • Olive oil, 1 tbsp. • Long grain rice, 1 cup • Curry spices, to taste
Total Energy	2512 Calories
Protein	154 g
Carbohydrate	312 g
Total Fat	75 g
*n-*3	4.4 g
ALA	1597 mg
EPA	300 mg
DHA	529 mg
Fibre (g)	58 g
Vitamin B12	3.4 μg
Iron	31.4 mg
Zinc	15.4 mg
Calcium	2226 mg
Iodine	173 μg
Vitamin D	34.5 μg

^a^Based on a 77 kg male gym goer; diet created using Nutritics (Nutritics Limited, Dublin, Ireland)

**Table 6:** Sample 3500 Calorie menu^a^

Meal	Ingredients
Breakfast: *“Tofu scramble with avocado toast”*	• Tofu, ½ block / 250 g • Whole-wheat bread, toasted, 3 large slices • Avocado, ¼, diced • Navel orange, 1 medium • 2 g microalgae oil supplement
Lunch / pre training: *“Roast vegetable, tempeh & buckwheat salad”*	• Buckwheat, raw, 1 cup • Tempeh, ¾ cup / 100 g • Zucchini, ½, roasted • Eggplant, ½, roasted • Onion, red, ½, roasted • Bell pepper, ½, roasted • Olive oil, balsamic vinegar, 1 tbsp. Each
Post-training snack: *“Spiced apple cereal”*	• Puffed rice, 2 cups • Apple, 1 large • Prunes, chopped, 18 g • Cinnamon, to taste • Soy milk, unsweetened, 270 ml • Rice protein, vanilla, 1.5 scoops / 35 g
Dinner: “*Spaghetti”*	• Black bean spaghetti, 75 g, raw weight • Spaghetti, dried, 88 g, raw weight • Pasta sauce, tomato, low sodium, ½ jar • Onion, red, ½ • Spinach, frozen, 70 g • Garlic, basil, to taste • Olive oil, 1 tbsp. • Nutritional yeast flakes (B12 enriched), 1 tbsp.
Snack: *“Chia, flax and hemp seed dessert”*	• Chia seeds, flax seeds, hemp seeds, 2 tbsp. Each • Almond milk, 1 cup • Dates, chopped, × 2 • Strawberries, ½ cup
Total Energy	3446 Calories
Protein	193 g
Carbohydrate	443 g
Total Fat	94 g
*n-*3	9.8 g
ALA	7754 mg
EPA	300 mg
DHA	529 mg
Fibre (g)	91 g
Vitamin B12	4.2 μg
Iron	42 mg
Zinc	40 mg
Calcium	2406 mg
Iodine	153 μg
Vitamin D	22.7 μg

^a^Based on a 94 kg male strength athlete; diet created using Nutritics (Nutritics Limited, Dublin, Ireland)

#### Vitamin D

Vitamin D is a fat-soluble vitamin produced in the skin, is essential for calcium absorption and bone health, and plays an important role in many physiological processes [117]. While humans synthesize vitamin D from exposure to sunlight, vitamin D can also be found in animal products and fortified foods [117]. Dietary intakes of vitamin D appear to be low in vegans who do not achieve sufficient sun exposure [118]. Cholecalciferol (D3) is an animal-derived version of vitamin D that is now widely available as a supplement [119]. Ergocalciferol [D2] is a vegan-friendly version of vitamin D but appears to be less bioavailable than cholecalciferol [119, 120]. Recently, however, vegan-friendly versions of cholecalciferol derived from lichen, a composite fungal-algae organism, have become commercially available, offering vegans a more bioavailable supplemental option. These supplements appear to be dosed similarly to animal-derived products, with dosages of 200–1000 IU per serving being common, and can be used as a like-for-like equivalent for animal-based counterparts.

In the USA, the IOM recommend an RDA of 600 I.U ∙ day^−1^ [117] for vitamin D. In the UK the Department of Health recommend 10 μg ∙ day^−1^ (400 I.U) is supplemented by individuals who do not achieve adequate sun exposure [121]. Of interest to athletes, Cannell et al. [122] suggest that optimising vitamin D status might improve athletic performance, if deficiency is present. Indeed, Moran et al. [123] highlight that poor vitamin D status negatively affects muscle strength and oxygen consumption, and suggest that supplementation might protect against overuse injury via its role in calcium metabolism and skeletal muscle function. Optimising vitamin D status is perhaps an important consideration for all athletes, regardless of dietary choice [124].

Effective vitamin D dosing might necessitate that supplementation is optimized via bespoke treatment strategies [125], based on individuals’ existing blood levels. In order to determine vitamin D status, plasma 25OHD levels can be sampled. Values <20 ng ∙ ml^−1^ are considered to be clinically deficient [126]. Optimal values might fall between 40 and 70 ng ∙ ml^−1^ [126, 127]. Recommendations for supplementation provided by Dalqhuist and colleagues [128] suggest that athletes should aim to achieve plasma 25OHD levels in the range of 30–40 ng ∙ ml^−1^. Dalqhuist and team [128] also suggest that supplemental doses of up to 4–5000 IU ∙ day^−1^ coupled with 50–100 μg ∙ day^−1^ of vitamin K1 and K2 could improve exercise recovery, allowing athletes to train more frequently. Data concerning performance-enhancing effects and toxicity at higher dosages of vitamin D have yet to be demonstrated, however a tolerable upper intake of 4000 IU ∙ day^−1^ has been established by the IOM [106]. Blood 25OHD values >150 ng ∙ ml^−1^ (plus hypercalcemia) are generally thought to signify toxicity [126]. Further research is warranted to determine optimal vitamin D doses for athletes.

### Supplements and ergogenic aids

#### Creatine

Research indicates that vegetarian and vegan diets reduce muscle creatine stores [129–131]. Creatine is a nitrogeneous, organic acid synthesized endogenously from arginine, glycine and methionine [132]. Foods such as meat, fish and poultry are rich sources of creatine but are excluded from a vegan diet. Creatine’s performance-enhancing effects have been well studied, and it appears that supplementation can improve short-term high-intensity exercise performance, muscle hypertrophy and maximal strength [132, 133]. Creatine supplementation might also lead to increased plasma volume, improved glycogen storage, improved ventilatory threshold, and reduce oxygen consumption during submaximal exercise [133]. Interestingly, data indicates that creatine supplementation might be *most* beneficial for athletes with low pre-existing muscle creatine stores. To highlight, Burke et al. [129] found that supplemental creatine attenuated low muscle creatine stores in vegetarians, who experienced greater improvements in FFM, maximal strength and type II muscle fibre area compared to omnivores. Creatine supplementation might therefore be an important ergogenic aid for vegan athletes to consider, and compensate for reduced muscle creatine stores experienced as a result of their lifestyle choices.

Dosing creatine effectively requires the achievement of muscle creatine saturation, and regimens of 20 g ∙ day^−1^ for 3–7 days to load creatine followed by maintenance doses of 3–5 g ∙ day^−1^ are common [132]. However, a smaller dose of 3–5 g ∙ day^−1^ taken over a 4-week period will achieve creatine saturation over the long term similarly [134]. Other protocols, such as 1 g ∙ 30 min^−1^ over 20 intakes per day, have also been suggested as means to achieve maximal saturation [135]. The co-ingestion of creatine with protein and carbohydrate might increase creatine retention by way of insulin-mediated storage, but appears not to have any noticeable performance-enhancing effects beyond stand-alone ingestion [133]. For vegan athletes who decide to supplement, powder forms of synthetic creatine are vegan-friendly (capsulated products might contain bovine gelatine), and the co-ingestion of creatine with whole food and/or a protein and carbohydrate mixture might be an optimal way of achieving creatine storage.

#### Beta alanine

Similar to muscle creatine levels, evidence also indicates that vegetarians have lower levels of muscle carnosine compared to omnivores [136, 137]. Carnosine, an intracellular proton buffer and antioxidant, is a cytoplasmic dipeptide (β-alanyl-l-histidine) found in skeletal muscle and the central nervous system, and is synthesised in situ from its rate-limiting precursor β-alanine [136]. Meat and poultry are the main sources of β-alanine in the diet, and β-alanine supplementation has been shown to increase muscle carnosine concentrations, leading to improvements in high-intensity exercise performance by way of buffering excess protons, scavenging free radicals, chelating transition metals and reducing fatigue [138, 139]. Achieving muscle carnosine saturation appears to be an important factor in effective β-alanine dosing, and research validates the efficacy of loading β-alanine in divided doses of 4–6 g ∙ day^−1^ for 2–4 weeks [139]. The efficacy of β-alanine supplementation has been confirmed in exercise >60 s duration, in aerobic exercise, and in attenuating muscle fatigue and improving time-trial performance in high-intensity exercise [139]. The effect of supplementation in exercise lasting <60 s is unclear however. Owing to muscle carnosine levels being lower in vegetarians than omnivores [137], it is feasible that the efficacy of β-alanine supplementation might also be augmented in vegans. Further research is necessary to validate this hypothesis however.

Taurine and β-alanine share transport mechanisms, meaning that supplemental β-alanine might theoretically inhibit taurine uptake in skeletal muscle [139, 140]. Taurine is a sulphur-containing amino acid that appears to play a role in many important physiological processes in humans, including bile acid conjugation, cardiovascular function, neurotransmission and euglycemia, and is obtained from seafood, meat and dairy products [140, 141]. Vegans have been shown to consume negligible amounts of taurine [142], which is conditionally essential in some clinical contexts [141]. It has been suggested that vegans might benefit from taurine supplements owing to its absence in the vegan diet [10]. However, further support for this recommendation could not be found in the literature located for this article. If indeed supplemental β-alanine does lead to reductions in taurine in humans, then vegans might be at greater risk of experiencing taurine depletion due to its absence from the diet. However, it must be noted that β-alanine has *not* been shown to reduce taurine levels in humans to date, and is considered to be safe when used within the parameters of recommended dosing [139].

The primary limitation of this review is the lack of research into veganism in sport. To mitigate this issue, information was gathered for this review from multiple sources, and inferences were made from the available data and via reasoned judgements. As such, many of the recommendations in this article require authentication, and so this article should serve as a catalyst for future research as well as a guidance document for athletes and practitioners. The main strength of this review is its comprehensiveness.

## Conclusions

In general, vegan diets tend to be lower in Calories, protein, fat, vitamin B12, *n*-3 fats, calcium and iodine than omnivorous diets, whilst concurrently being higher in carbohydrates, fibre, micronutrients, phytochemicals and antioxidants. Achieving a high energy intake is difficult in some instances, owing to plant-based foods promoting satiety. Issues with the digestibility and absorption of nutrients such as protein, calcium, iron and zinc might be an issue too, meaning that athletes might need to consume higher amounts of these foods compared to omnivores and other vegetarians. However, through the strategic selection and management of food choices, and with special attention being paid to the achievement of energy, macro and micronutrient recommendations, along with appropriate supplementation, a vegan diet can achieve the needs of most athletes satisfactorily. Supplementation with creatine and β-alanine might offer augmented performance-enhancing effects in vegans, who experience low pre-existing levels of these substances, and further research is needed to investigate the performance-enhancing effects of these substances in vegan populations. For some, a vegan diet is the manifestation of important ethical beliefs, and requires diligence to sustain [5–7]. It is a central tenet of this article that similar conscientiousness needs be paid to achieving dietary sufficiency, otherwise health and performance could suffer over the long term if an individual’s nutrition is not managed appropriately.

## Abbreviations

25OHD: 25-hydroxyvitamin D
ALA: α-linolenic acid
AND: Academy of Nutrition and Dietetics
BCAA: Branched Chain Amino Acid
DHA: Docosahexaenoic acid
DIAAS: Digestible Indispensible Amino Acid Score
DRI: Dietary Reference Intake
EPA: Eicosapentaenoic acid
FFM: Fat Free Mass
IOM: Institute of Medicine
ISSN: International Society of Sports Nutrition
PDCAAS: Protein Digestibility Corrected Amino Acid Score
RDA: Recommended Daily Allowance
TSH: Thyroid-Stimulating Hormone
